# Canine Retina Has a Primate Fovea-Like Bouquet of Cone Photoreceptors Which Is Affected by Inherited Macular Degenerations

**DOI:** 10.1371/journal.pone.0090390

**Published:** 2014-03-05

**Authors:** William A. Beltran, Artur V. Cideciyan, Karina E. Guziewicz, Simone Iwabe, Malgorzata Swider, Erin M. Scott, Svetlana V. Savina, Gordon Ruthel, Frank Stefano, Lingli Zhang, Richard Zorger, Alexander Sumaroka, Samuel G. Jacobson, Gustavo D. Aguirre

**Affiliations:** 1 Department of Clinical Studies, School of Veterinary Medicine, University of Pennsylvania, Philadelphia, Pennsylvania, United States of America; 2 Department of Ophthalmology, Scheie Eye Institute, Perelman School of Medicine, University of Pennsylvania, Philadelphia, Pennsylvania, United States of America; 3 Department of Pathobiology, School of Veterinary Medicine, University of Pennsylvania, Philadelphia, Pennsylvania, United States of America; 4 Department of Biochemistry, School of Dental Medicine, University of Pennsylvania, Philadelphia, Pennsylvania, United States of America; 5 Department of Neuroscience, Perelman School of Medicine, University of Pennsylvania, Philadelphia, Pennsylvania, United States of America; National Eye Institute, United States of America

## Abstract

Retinal areas of specialization confer vertebrates with the ability to scrutinize corresponding regions of their visual field with greater resolution. A highly specialized area found in haplorhine primates (including humans) is the *fovea centralis* which is defined by a high density of cone photoreceptors connected individually to interneurons, and retinal ganglion cells (RGCs) that are offset to form a pit lacking retinal capillaries and inner retinal neurons at its center. In dogs, a local increase in RGC density is found in a topographically comparable retinal area defined as the *area centralis*. While the canine retina is devoid of a foveal pit, no detailed examination of the photoreceptors within the *area centralis* has been reported. Using both *in vivo* and *ex vivo* imaging, we identified a retinal region with a primate fovea-like cone photoreceptor density but without the excavation of the inner retina. Similar anatomical structure observed in rare human subjects has been named fovea-plana. In addition, dogs with mutations in two different genes, that cause macular degeneration in humans, developed earliest disease at the newly-identified canine fovea-like area. Our results challenge the dogma that within the phylogenetic tree of mammals, haplorhine primates with a fovea are the sole lineage in which the retina has a central bouquet of cones. Furthermore, a predilection for naturally-occurring retinal degenerations to alter this cone-enriched area fills the void for a clinically-relevant animal model of human macular degenerations.

## Introduction

Vertebrate retinas have evolved over several hundred million years to use exquisitely organized and interconnected neuronal layers to capture the spatial distribution of photon flux from the visual environment, integrate and process it, and signal to higher visual centers [1], [2]. There are six major types of neurons, the spatial densities of which tend to change gradually across the retina. But there are also abrupt changes in neuronal density. The best studied retinal region with such an abrupt change is the *fovea centralis* (or ‘fovea’ for short) named after the distinct pit formed in the central retina by centrifugal movement of inner retinal layers and photoreceptor synapses during development [3]. Foveas have been identified, since at least the early part of the 19^th^ century, in most primates, including humans, and some reptiles, birds and fish [4], [5]. A fovea usually contains multiple rows of highly packed cone photoreceptors resulting in the highest density across a given retina [6], [7]. In old-world primates, foveal cone photoreceptors are connected through elongated axons to bipolar cells which in turn connect to ganglion cells to form private pathways to the visual cortex [8]. This connectivity pattern implies the existence of a high spatial density of post-synaptic neurons which ‘belong’ to the fovea even though they are physically located in an annulus around the fovea as a result of pit formation [9]. Overall, the location of the fovea (temporal to the optic axis of the eye), the peak density of cone photoreceptors therein, and their connectivity support the hypothesis that this small retinal region is specialized for high acuity binocular vision [10].

In non-primate mammals, there is a central region of specialization called *area centralis* which also is often located temporal to the optic axis and demonstrates a local increase in photoreceptor and retinal ganglion cell (RGC) density [5], [11]–[17]. Extensive studies in cats have reported increased density of RGCs in the *area centralis* [18]–[21] with evidence for centrifugal displacement of their soma in relation to their dendritic tree [22] which would be expected to reduce the thickness of the RGC layer in this area of higher visual acuity; however, cats are not known to have the degree of cone photoreceptor packing found in the primate fovea. Yet, the correspondence between visual fixation and *area centralis* supports the hypothesis that among vertebrates, the *fovea centralis* and the *area centralis* are part of a spectrum of structural specializations that share similar features to optimize visual acuity [10]. This claim is strengthened by the reports of relatively high and comparable cone densities in the *area centralis* of other mammals [12], [23] including that of the dog [17]. With an increasing number of identified naturally-occurring genetic mutations that are also associated with retinal degeneration in humans, the dog is proving to be a valuable large-animal model for the understanding of the pathogenic mechanisms of these diseases, and the preclinical testing of novel therapeutic approaches [24], [25]. Detailed characterization of the canine *area centralis* and its susceptibility to diseases is, however, currently lacking.

In the present study, we used state of the art non-invasive, and histological imaging to examine the canine *area centralis* and discovered at its center, a primate fovea-like area of extreme subspecialization comprising a unique packing of cone photoreceptors not previously described in any non-primate mammalian species. Furthermore, dogs carrying mutations in two genes that cause maculopathies in humans, had a predilection for early disease in this cone-enriched area.

## Materials and Methods

### Animals and Ethics Statement

All dogs were bred and maintained at the University of Pennsylvania Retinal Disease Studies Facility (RDSF), Kennett Square, PA, and supported by facility grants from FFB and NEI/NIH EY06855. The studies were carried out in strict accordance with the recommendations in the Guide for the Care and Use of Laboratory Animals of the National Institutes of Health, the USDA's Animal Welfare Act and Animal Welfare Regulations, and complied with the ARVO Statement for the Use of Animals in Ophthalmic and Vision Research. The protocols were approved by the Institutional Animal Care and Use Committee of the University of Pennsylvania (IACUC Protocol #s 803813, 801870, 803422). All non-invasive imaging procedures were performed under general (isopentane) gas anesthesia, ocular tissues were collected after euthanasia with intravenous injection of euthanasia solution (Euthasol;Virbac, Ft. Worth, TX), and all efforts were made to improve animal welfare and minimize discomfort.

Included were 24 normal (wildtype, WT) dogs (ages: 2 weeks –8 years), 7 *BEST1*-mutant dogs (ages: 9–112 weeks), and 14 *RPGR*-mutant dogs (ages: 2–145 weeks). *BEST1*-mutant dogs were either homozygous for the c.73C>T stop allele (n = 5), homozygous for two linked mutations c.[1388delC; 1466G>T] that are arranged in cis configuration (n = 1), or were compound heterozygous for the stop and linked mutations (n = 1) of the bestrophin gene (GeneBank accession no. EF110978). *RPGR*-mutant dogs had either the c.1084-1085delGA (n = 9), or the c.1028-1032delGAGAA (n = 5) allele in exon ORF15 (GeneBank accession no. AF385621) of the *RPGR* gene. Genotyping of the dogs has been previously described [26]–[28]. All dogs had a mesocephalic head conformation which was confirmed by calculating the cephalic index [(skull width/skull length)*100] [29].

### *In vivo* Retinal Imaging

Non-invasive *en face* and cross sectional imaging was performed by confocal scanning laser ophthalmoscopy (cSLO) and spectral domain optical coherence tomography (SD-OCT) after pupillary dilation and under general anesthesia [30]–[32]. Overlapping *en face* images of reflectivity with near-infrared illumination (820 nm) were obtained (HRA2 or Spectralis HRA or Spectralis HRA+OCT, Heidelberg Engineering GmbH, Germany) with 30° and 55° diameter lenses to delineate fundus features such as optic nerve and retinal blood vessels. In a subset of eyes, short-wavelength (488 nm) illumination was used to image autofluorescence. Custom programs (MatLab 7.5; The MathWorks, Natick, MA) were used to digitally stitch individual photos into a retina wide panorama. SD-OCT was performed with line and raster scans (RTVue-100, Optovue, Inc. Fremont, CA or Spectralis HRA+OCT). Overlapping raster scans covered large regions of the retina. Either 6×6 mm (101 lines of 513 longitudinal reflectivity profiles (LRPs) each, no averaging, Optovue) or 9×6 mm (49 lines of 1536 LRPs each, averaging 8–10, Spectralis) was used. Axial length was measured (Sonomed A-scan A1500, Sonomed-Escalon, Lake Success, NY) after each imaging session.

Post-acquisition processing of OCT data was performed with custom programs (MatLab 7.5). For retina-wide topographic analysis, integrated backscatter intensity of each raster scan was used to locate its precise location and orientation relative to retinal features visible on the retina wide mosaic formed by NIR reflectance images. Individual LRPs forming all registered raster scans were allotted to regularly spaced bins (1°×1°) in a rectangular coordinate system centered at the optic nerve; LRPs in each bin were aligned and averaged. Intraretinal peaks and boundaries corresponding to histologically definable layers were segmented semi-automatically with manual override using both intensity and slope information of backscatter signal along each LRP. Specifically, the retina-vitreous interface, outer plexiform layer (OPL), outer limiting membrane (OLM), signal peak near the inner/outer segment (IS/OS) junction, and the retinal pigment epithelium (RPE) were defined. In the superior retina of the dog, backscatter from the tapetum forms the highest intensity peak, and RPE and IS/OS peaks are located vitreal to the tapetal peak. In the inferior retina, backscatter from the RPE forms the highest intensity peak. ONL thickness was defined from the sclerad transition of the OPL to the OLM, and ONL thickness topography was calculated. For all topographic results, locations of blood vessels and the optic nerve head were overlaid for reference.

### Retinal magnification factor (RMF)

Lateral distance on the retina corresponding to the angular pivot of a scanning laser imaging beam depends on the RMF for each eye. RMF (and other optical characteristics) of individual eyes can be calculated from simplified schematic eyes if relevant biometric parameters are known or measurable [19], [33]. We used a simpler alternative which is based on the estimate of the posterior nodal distance (PND) and the formula RMF = 2*π*PND/360, where RMF is given in mm/deg and PND is in mm [34]. PND in turn was estimated as a fixed proportion (59%) of the axial length of the eye for dogs [33], [34]. Axial length was individually measured and ranged from ∼15 mm in young dogs (7 wks) to ∼21 mm in adults; and the corresponding RMF ranged from 0.15 to 0.22 mm/deg similar to a previous report of 0.22 mm/deg in adult dogs [33].

### Microscopic morphology

#### Confocal microscopy imaging of RGCs on retinal wholemounts

The retinal wholemount technique was used to image RGCs, and cones in 5 dogs (8 eyes). Following humane euthanasia by intravenous injection of a euthanasia solution (Euthasol; Virbac, Ft. Worth, TX) the eyes were immediately removed and neuroretinas were separated from the RPE, fixed in 4% paraformaldehyde for 4 hours at 4°C, blocked in normal goat serum and incubated for 3–4 days at 4°C in a solution composed of either rhodamine-labeled peanut agglutinin (PNA; 1/1,000, Vector labs) or human cone arrestin antibody (LUMIf; 1/10,000 provided by C. Craft, University of Southern California) to label cones, von Willebrand factor VIII antibody (A0082, 1/400; Dako, Carpinteria, CA) to label the retinal vasculature, and Brn3a antibody (MAB1585;1/50; Millipore, Billerica, MA) to label RGCs. Fluochrome-labeled secondary antibodies (Alexa Fluor; 1/200, Molecular Probes, Grand Island, NY) with DAPI nuclear stain, were applied for 24 hours at 4°C. A Nikon A1R laser scanning confocal microscope (Nikon Instruments Inc., Melville, NY) equipped with multi Argon 488 and 561 DPSS lasers was used to image the RGCs and the retinal vasculature throughout the entire surface of the retina. The creation of a large image mosaic was made by defining inferior, superior, temporal and nasal boundaries of the sample and the stitching and blending together of the individual 512 μm ×512 μm image fields captured from a Plan APO 10 X Objective (NA 0.45). During acquisition, a 9×10 μm capture along the Z axis was coordinated with the pre-defined XY coordinate stage movement. To correct for any variation in specimen thickness, a focus surface map was created of the retina. These coordinates of pre-focused points in X, Y and Z planes allowed for a smooth interpolation surface for a neighboring non-focused point thereby offering greater fidelity during image capture in the Z plane. Image Capture and Analysis was done using Nikon Elements Software 4.0.

Post acquisition retinal ganglion cell segmentation was performed by first thresholding the image, and applying restrictions for object area of 25∶250 pixels, mean intensity, and circularity range of 0.19∶1.0. The cell locations were then analyzed using the Python NumPy and MatPlotLib libraries. The cell coordinates were inserted into a matrix and counted over sectional areas of 100 μm by 100 μm, which was then placed in a mesh grid and plotted as a three dimensional surface with a spectral color density map.

#### Full thickness retinal imaging by two-photon microscopy

Following identification of the site of peak RGC density, 2-photon microscopy was used to enable full-thickness imaging of a retinal area of 771 μm by 771 μm to identify the cones located on the opposite (distal) side of the peak of RGC density. Multi-photon images were acquired on a Leica TCS SP5 2-Photon system with a 20 X (1.0 NA) water immersion objective lens and a Coherent Chameleon Ultra II Ti:Sapphire pulse laser tuned to 800 nm (Coherent Inc., Santa Clara, CA). External PMT detectors were used. Z-planes were taken at intervals of 0.99 µm in 1024 ×1024 pixel format at 400 Hz with a line average of 4. PMT-based compensation was utilized to adjust gain settings during Z-stack acquisition. A single Z scan within the plane of the cone IS was then used to manually count cones in a 50 μm ×50 μm window within the region of highest cone concentration, and cell density was calculated. Two-photon microscopy images were imported into Volocity and/or Camtasia Studio for 2D and 3D visualization.

#### Retinal cross sections

A subset of dogs had the eyes processed for morphological evaluation on retinal cryosections as previously described [35]. Each eye had 100 to 250 serial 10-μm-thick cryosections cut within the temporal superior quadrant that included the entire area centralis, and every tenth section was H&E stained to locate the region that comprised multiple rows of RGCs. Within that region, the area of peak cone density was identified by performing immunohistochemistry on adjacent sections with antibodies directed against human cone arrestin, rod opsin (MAB5316; 1/200, Millipore), RPE65 (PETLET; 1/1,000 provided by T. M. Redmond, NEI/NIH, Bethesda, MD), and DAPI to label cell nuclei. Sections were imaged either by widefield epifluorescence (Axioplan; Carl Zeiss Meditec GmbH Oberkochen, Germany) or confocal microscopy (Leica TCS SP5; Leica Microsystems CMS GmbH, Mannheim, Germany). Sections that included the area of peak cone packing were used to estimate the cone density by using the following formula [36]:
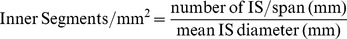



Cone IS were counted over a 50 μm retinal length centered on the region with maximal number of rows of cone somata.

In addition, archival epoxy resin (Epon) embedded retinal tissues of *RPGR* mutant dogs that included the *area centralis*, were cut (1 μm thickness) and stained as previously described [35] with azure II-methylene blue and a paraphenylenediamine counterstain. This staining technique provides good visualization/distinction of rods versus cones. The region of peak cone density was similarly identified.

#### Comparison of peak cone densities of wildtype dogs to that of macaques and humans

Peak cone densities of dogs measured on retinal wholemounts or estimated from counts of inner segments on retinal cross sections were compared to published values of cone densities in the fovea of macaques [6], [37], [38], as well as that of humans measured by adaptive optics [39]–[41], or histology [42]–[44].

### Comparison of retinal distances *in vivo* versus microscopic morphology

Several methods were used to estimate the relationship between retinal distances *in vivo* with those in tissue processed for morphology. First considered was the histology fixation and embedding protocol that was used for obtaining retinal cross sections. We took a 13 mm circular biopsy punch of a wildtype dog eye which included the retina, choroid and sclera within 2 min after enucleation and photographed it with a caliper. The punch was then immediately processed for fixation in paraformaldehyde, cryoprotected in solutions of sucrose in phosphate buffered saline, and embedded in optimal cutting temperature mounting medium following the same protocol as indicated above for retinal cryosections [35]. Following embedding, cross-sectioning was done until the diameter of the punch was reached. The length of the section was measured and found to be nearly exactly 13 mm suggesting a lack of substantial shrinkage/expansion of the processed tissue compared to the fresh tissue. Second, we directly compared retinal distances measured *in vivo* with the same distances measured on histological sections in the same eyes. The distance measured for this purpose *in vivo* was between the center of the localized ONL thinning supero-temporal to the ONH and the edge of the ONH. For histology, H&E stained cross-sections that included both the region of cone packing and at least a portion of the ONH were used and the distance between these two landmarks was measured. The comparison showed the histological distance to be on average 4% greater than the in vivo distance (range −7.2% to +10.3%, n = 9 eyes of 8 dogs). And third, we estimated the magnification required to register the blood vessel pattern imaged *in vivo* with the blood vessel pattern of a retinal whole mount that was processed as described above. The comparison showed the histological distance to be on average 6.5% shorter than the *in vivo* distance (range −13.8% to +3.5%, n = 4 measurements). We conclude that *in vivo* retinal distances are within 10% of histological measurements and thus all histological measurements are presented without correction.

### Statistics

Descriptive statistics including mean and SD were used respectively as a measure of central tendency, and deviation from the mean, and used to report 95% confidence intervals.

## Results

**Figure 1:**
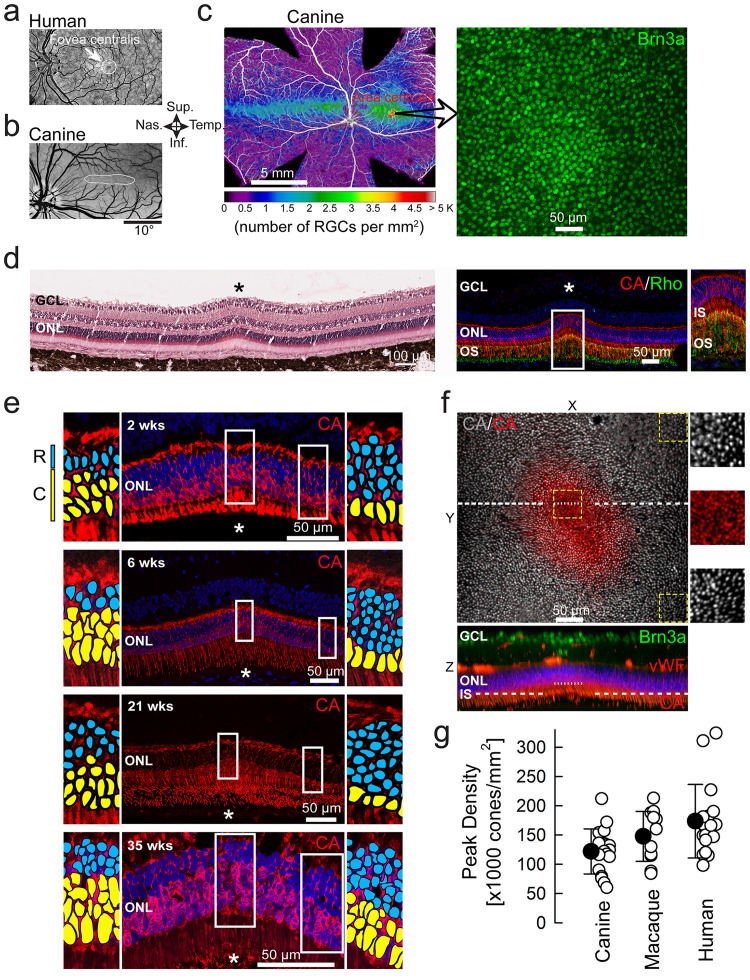
High density of cone photoreceptors at the wildtype canine fovea-like area. (a,b) Central areas of specialization (white) are avoided by retinal blood vessels in human and canine eyes. (c) Retinal ganglion cell (RGC) density map across the canine retina and peak density at the center of the area of specialization. Brn3a: brain-specific homeobox/POU domain protein 3A (d) Retinal cross-section (H&E stained) through the fovea-like area of a 6 week-old dog shows a focal elevation on the retinal surface, thickening of the ganglion cell layer (GCL), and thinning of the outer nuclear layer (ONL). Immunohistochemistry at 7 weeks shows focal high density of cones (red), markedly reduced density of rods (green), elongated inner segments (IS), outer segments (OS) and multiple layers of RGCs in GCL. CA: cone arrestin; Rho: Rhodopsin. ONL is stained with DAPI (blue). (e) Abrupt increase of cone density associated with an abrupt decrease of rod density in 4 eyes (at different postnatal ages for later comparison with mutant dogs). Rod and cone nuclei are highlighted in enlarged insets with blue and yellow, respectively, for visibility. (f) Two-photon microscopy imaging of the fovea-like area and immediate surrounding region. *En face* view (XY) is an overlay of two Z scans taken at different depths (shown as dotted lines on the orthogonal XZ view) to illustrate the cone IS densities at the fovea-like area and surrounding regions. Insets illustrate the abrupt increase in central peak cone IS density within the canine fovea-like area. vWF: von Willebrand factor VIII; (g) Comparison of peak cone densities in dogs to that reported for macaques and humans measured by adaptive optics imaging, or histology Filled symbols are mean±sd.

Similar to the human *fovea centralis* (**Fig. 1a**), the canine *area centralis* is found within a region that is avoided by large retinal vessels (**Fig. 1b**) and can be readily identified histologically on retinal wholemounts labeled with a marker for RGCs (**Fig. 1c**). Upon serial sectioning through the entire *area centralis*, we encountered a small region with distinctive morphological features including multiple (2.8±0.3; n = 11) rows of RGCs, localized thinning of the outer nuclear layer (ONL), and discrete elevation of the retinal surface (**Fig. 1d** **, left**). At this site, immunohistochemistry revealed thin and extended cone inner segments as well as elongated cone and rod outer segments (**Fig. 1d** **, right**); the basis of the localized thinning of ONL was a substantial reduction in the number of rows of rods (from ∼8 to 2.6±1.2; n = 11) but there was an abrupt increase (from ∼1 to 2.9±0.6; n = 11) in the number of rows of densely packed cone somata (**Fig. 1e**).

The spatial density of cone photoreceptors was further characterized by two-photon microscopy using markers specific to cone photoreceptors (cone arrestin antibody) or their insoluble extracellular matrix domain (peanut agglutinin) to image individual inner segments in tangential section (**Fig. 1f**). There was a small area (15,500±5,800 μm^2^; n = 5) of densely packed cone inner segments (**Fig. 1f**; **Videos S1 & S2**) similar to that seen at the center of the human fovea called “foveola” where elongated and slim cone IS are found [6]. Wholemount examinations of 7 additional canine eyes and cross-sections in 11 eyes showed peak cone densities to range from 64K to 212K cells/mm^2^ with an average of 127 (±40) K cells/mm^2^ (n = 15; **Table S1**) which is at least 5 fold higher than previously reported [17], similar to macaque [6], [37], [38], and overlaps with the range published for the human fovea [39]–[44] (**Fig. 1g**). When adjusted for the retinal magnification, inter-cone distances subtended a visual angle of 0.75 min arc similar to that in humans [7]. Interestingly, the peak cone density variation between dogs was also similar to the substantial inter-individual variability described in human foveas [43]. Racial differences across humans suggest a wide natural spectrum of foveal anatomy [45] and at one extreme of this spectrum is a variant, called fovea-plana, identified in individuals lacking excavation of inner retina but retaining high cone density and good visual acuity [3]. We propose that the discovery within the canine *area centralis* of this previously unknown region of high cone density makes it the equivalent to the outer retina of the human foveola.

**Figure 2:**
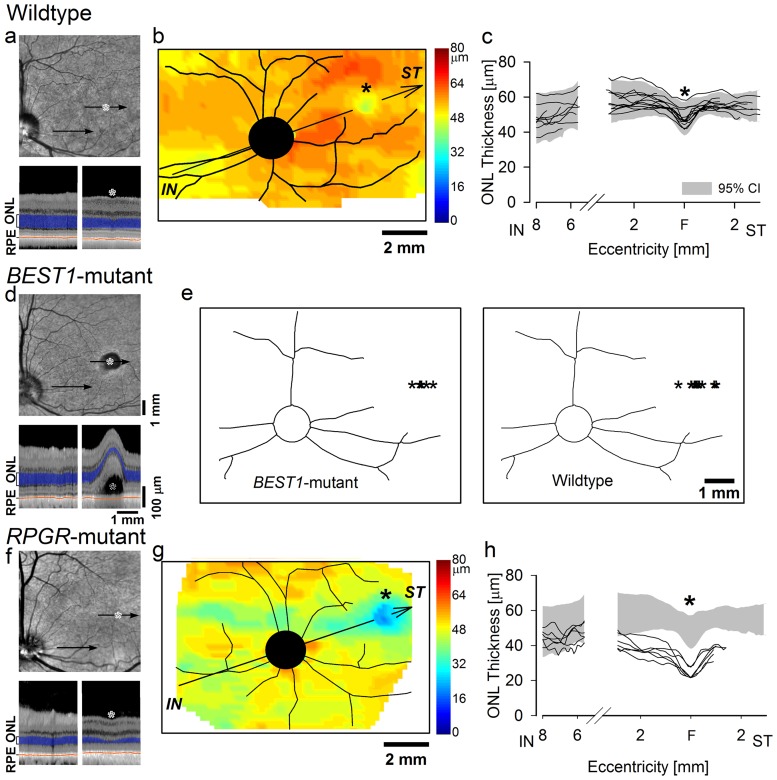
Photoreceptor layer lamination in wildtype dogs and in naturally-occuring genetic diseases primarily affecting the canine fovea-like area. (a,d,f) *En face* infrared view of representative wildtype (a), *BEST1*-mutant (d) and *RPGR*-mutant (f) dogs. *, fovea-like area. Arrows, locations of cross-sectional OCT scans shown below each panel. Outer photoreceptor nuclear layer (ONL) and retinal pigment epithelium (RPE) are highlighted for visibility on OCT scans. (b) ONL thickness topography in a 22-wk-old wildtype dog displayed in pseudo-color. There is a distinct localized region of ONL thinning supero-temporal (ST) to the optic nerve head (black circle) corresponding to the fovea-like area. Major blood vessels are overlaid. (c) Diagonal profiles of ONL thickness (along arrow shown in b) in individual wildtype dogs (lines; ages: 7 wks –8 yrs; n = 13). 95% confidence interval shown (gray area). The break in the axis corresponds to the optic nerve head which lacks photoreceptors. F, fovea-like area. (e) Topographic localization of the sites (*) of the early macular lesions in *BEST1*-mutant dogs (ages: 10–62 wks; n = 7, left) correspond to the localization of the fovea-like area in wildtype dogs (ages: 7 wks –8 yrs; n = 13, right). (g) ONL thickness topography in an 11-wk-old *RPGR*-mutant dog displayed in pseudo-color. *, fovea-like area. (h) Diagonal profiles of ONL thickness in young *RPGR*-mutant dogs (lines; age: 11 wks; n = 6 eyes of 3 dogs) shows abnormal thinning corresponding to the fovea-like area and its immediate surrounds compared to WT (gray area). All eyes are shown as left eyes (temporal retina to the right).

Devastating and untreatable vision loss of children and young adults are caused by certain gene mutations affecting the photoreceptors of the fovea and the surrounding region called the macula [46]. In addition, foveas in the elderly are commonly affected with age-related macular degeneration (AMD), a multifactorial disease with a strong genetic component [47]. Faithful modeling of human maculopathies in mice is challenging because mice lack a fovea/macula, and genetic manipulations in primates are not practical. Discovery of canine maculopathies genetically and anatomically matched to the human disease could have important consequences for the development of therapies [24]. We first used *in vivo* microscopy with OCT to define the properties of the wildtype canine fovea-like area in life (**Fig. 2a**). Mapping of the thickness of the ONL layer showed an area with distinct thinning located supero-temporal to the optic nerve head (ONH) (**Fig. 2b–c**). The distances from the region of ONL thinning to the center or the edge of the ONH averaged 4.35 (±0.33; n = 13) or 3.53 (±0.34; n = 13) mm, respectively. On histologic serial sectioning, the distance between the peak cone density and the edge of the ONH was 3.8 (±0.30; n = 15) mm (**Table S1**). These results provided the opportunity to evaluate the canine fovea-like area *in vivo* in health and disease.

**Figure 3:**
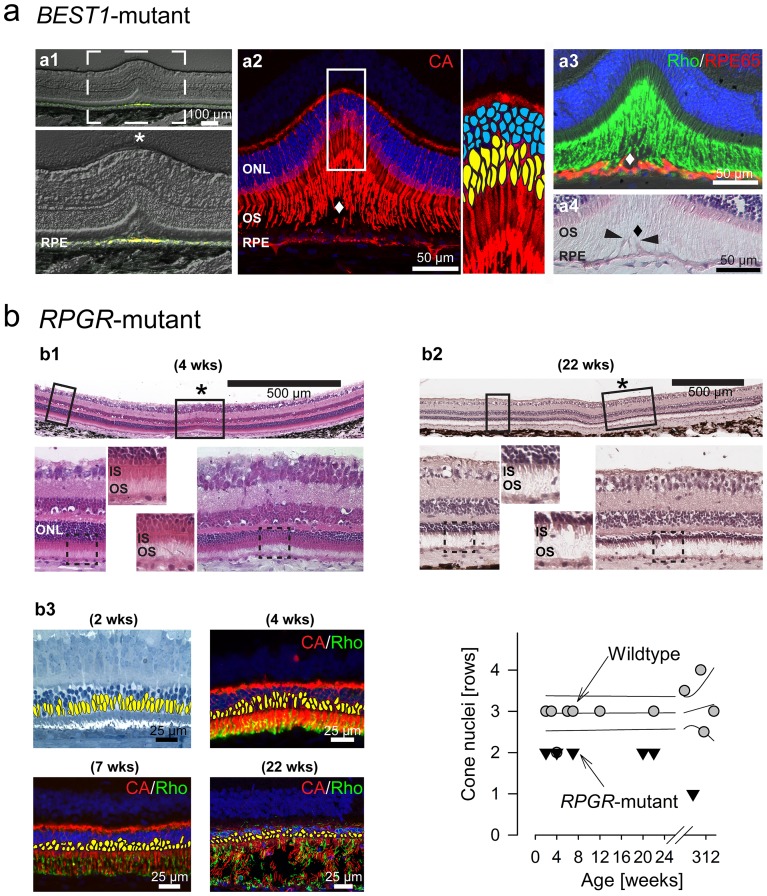
Histolopathology at the fovea-like area in two canine models of inherited macular degeneration. (a) Fovea-like area in a 112 week-old *BEST1*-mutant dog. (a_1_) Epifluorescence microscopy image (with DIC/Nomarski optics) showing at the fovea-like area increased autofluorescence (yellow) in the retinal pigment epithelium (RPE). (a_2_) Immunohistochemistry on the same section as (b_1_) shows focal separation (diamond) of cone (red) outer segments (OS) from the underlying RPE. Note: Red fluorescent signal originating from the RPE is endogenous autofluorescence (see a_1_). (a_3_) Immunohistochemistry shows focal separation (diamond) of rod OS (green) from hypertrophied RPE cells (red; arrowheads), and (a_4_) extension of RPE apical processes (arrowheads). (b) Fovea-like areas in *RPGR* mutant dogs. (b_1_) Horizontal retinal cross-section (H&E stained) shows the abrupt ONL thinning and shortened inner segments (IS) at the fovea-like area of a 4 week-old mutant dog while ONL thickness and structure of photoreceptors is preserved in the immediate peri-foveal regions. (b_2_) Horizontal retinal cross-section (H&E stained) shows more prominent ONL thinning at the fovea-like area which has now extended peri-foveally in a 22 week-old dog. (b3) Early reduction in the number of cones is seen at the fovea-like area on retinal cross sections (left; cone nuclei are highlighted in yellow for visibility) and quantitative comparison to wildtype results (right). CA: cone arrestin; Rho: rhodopsin.

Mutations in the *BEST1* gene cause human maculopathy by detaching photoreceptors from the RPE [48]. We examined dogs with naturally-occurring *BEST1* mutations [27]; at earliest ages, there were no detectable retinal abnormalities whereas in older dogs there were retina-wide abnormalities. Between 10 and 62 weeks of age, a single focal lesion (**Fig. 2d** **top**) appeared in one or both eyes in the supero-temporal retina at 4.66 (±0.33; n = 7) mm to the center of the ONH; the location of the *BEST1* lesions corresponded to the wildtype canine fovea-like area (**Fig. 2e**). On cross-sectional imaging, the lesion involved a local separation of the retina from the RPE (**Fig. 2d** **lower right**). With time, the retinal separation frequently enlarged, subretinal deposits formed which showed abnormal increases in autofluorescence at this site as well as in extrafoveal locations (**Fig. S1; Table S2**). Histology at the fovea-like area (**Fig. 3a**) showed abnormal accumulation of autofluorescent material in the RPE, and confirmed the retinal separation (**Fig. 3a** **_1_**). This was associated with a disruption of the close contact between the outer segments of foveal cones (**Fig. 3a** **_2_**), rods (**Fig. 3a** **_3_**) and the underlying RPE whose apical processes were elongated (**Fig. 3a** **_4_**).

Mutations in *RPGR* gene cause a human photoreceptor ciliopathy with a wide phenotypic spectrum that often involves a strong macular disease component [49]. We examined dogs with a naturally-occurring mutation in *RPGR* [35]. The earliest disease detectable non-invasively consisted of an abnormal thinning of the ONL at the fovea-like area (**Fig. 2f–h**). This was confirmed by histology (**Fig. 3b**) as early as 2–4 weeks of age, while ONL thickness and photoreceptor structure at perifoveal locations were within the normal range (**Fig. 3b** **_1_**). At later ages (**Fig. 3b** **_2_**), central disease along the visual streak followed by retina-wide photoreceptor loss was seen as previously reported [31]. Thinning of the ONL at the fovea-like area was initially due to reduced packing of cones (**Fig. 3b** **_3_, left**) followed by a loss of rods and cones (**Fig. 3b** **_3_, right; and Table S3**).

## Discussion

Our discovery of extreme cone photoreceptor enrichment near the central vision area of dogs that resembles the primate fovea highlights the overlap in retinal structure in two distinct phylogenetic trees. The canine fovea-like area shares not only anatomical features with the primate fovea, but also similar susceptibility to genetic damage. Such commonalities support the use of the canine retina as a model system to understand the development and maintenance of the normal foveal photoreceptors, their response to genetic disease, and evaluation of potential treatments that could be applicable to human macular degenerations.

### Extreme sub-specialization within the *fovea centralis*: Foveola and the central bouquet of cones

The human *fovea centralis* covers about 0.2% of the total retinal area, similar to other primates [50]. Curiously, within this already tiny *fovea centralis* is an even smaller region (∼200 µm in diameter) of extreme sub-specialization called foveola where cone photoreceptor densities double over a 100 µm distance [37], [43], [44] giving rise to a central ‘bundle’ or ‘bouquet’ of about 500 cones [44], [51]. The foveola is also the region that lacks rods and retinal capillaries [37], [43], [52]. The foveola corresponds to the ‘bottom’ of the pit formed by the *fovea centralis* and it has been hypothesized [53] that the mechanical forces involved in the pit formation also contribute significantly to the cone photoreceptor packing of the foveola [6], [37]. Consistent with this hypothesis would be the apparent lack of cone packing in the cat without a foveal pit [12], [18]. Inconsistent with a direct link between pit formation and cone packing is the substantial displacement between *fovea centralis* and the highest cone density region in owl monkeys and prosimian bush-babies [38]. In addition, rare individuals with oculocutaneous albinism show a variant of central retinal specialization named fovea-plana with cone packing that can reach densities of the normal foveola and result in normal visual acuity without pit formation [3], [54], [55]. Furthermore, it appears that the fovea-plana may be one extreme of a wide variation of foveal pit morphology observed across normal humans [45]. Our findings of a small region near the temporal side of the canine *area centralis* with dramatic evidence of cone packing reaching densities similar to primate foveola presents yet another challenge to the hypothesis that links pit formation to cone packing, and supports the hypothesis that there is continuity of retinal structural specialization between *fovea centralis* and *area centralis* [10].

### Cone photoreceptor packing and visual acuity

The spatial resolving capacity of the visual system is dependent on optical and neural factors. Neural factors include cone photoreceptor, interneuron, and retinal ganglion cell densities in the areas of retina specialization, magnification factor of the primary visual cortex, and receptive fields of neurons along the visual system [56]. The extreme packing of cones in the primate foveola coupled to low cone to retinal ganglion cell (RGC) convergence supports the ability of the eye to spatially discriminate two points that are close together. The normal human eye is able to resolve lines that are separated by a visual angle smaller than 1 arcminute, which is equivalent to 30 cycles per degree (cpd), or 20/20 vision on the Snellen chart. In the canine fovea-like area, cone densities in the 60–212K cones/mm^2^ range should theoretically support a visual resolution of 25–46 cpd (20/24-20/13 Snellen acuity), assuming sufficiently low optical aberrations and low convergence of cones onto RGCs. However, the canine visual acuity is estimated to be no better than 12.6 cpd (20/50 Snellen acuity) [57]–[61]. This apparent mismatch could be due to limitations of performing visual acuity measures under non-optimum conditions [62].

An alternative hypothesis includes the light scattering. Canids, as well as several other vertebrates, have evolved a retinal region with amelanotic RPE cells and a reflective *tapetum lucidum* layer between the non-pigmented RPE cells and the large choroidal blood vessels [63]; the canine fovea-like area is located within this tapetal region. The lack of light-absorbing pigment together with a mirror-like reflecting surface is thought to maximize photon capture to increase retinal sensitivity for nocturnal vision. However, this advantage comes at the cost of reduced acuity resulting from scattered photons stimulating photoreceptor cells neighboring the initial ray of light. The effect of scattered photons would be minimal however near the cone photoreceptor threshold and thus we speculate that the packing density of cones at the canine fovea-like area may allow the necessary visual acuity for canids to hunt under twilight conditions. Testing such a hypothesis, would require measuring visual acuity in dogs under low light ambient conditions.

### Location of the canine fovea-like area

The approximate location of the *area centralis* in the dog can be estimated ophthalmoscopically by observing the convergence of arterioles and venules towards an area supero-temporal to the optic nerve head. For the precise non-invasive localization of the canine *area centralis*, we took advantage of the thinning of the outer nuclear layer near the center of the *area centralis* [11], [12], [64]. The center of ONL thinning was 22° (4.35 mm) supero-temporal to the center of the optic nerve head (n = 11, ages 7 to 247 wks). This was consistent with morphological appearance of cone packing and canine fovea-like area at the same distance in a subset of eyes where both imaging and histology were available. There was a tendency for the younger eyes with shorter axial globe lengths to have a larger angular distance to the locus of ONL thinning, similar to that shown in developing kittens [21]. Our estimate of the location of the canine fovea-like area located within the *area centralis* is similar to many previous estimates of the center of the *area centralis* [11], [16], [65]. Curiously, a substantially different eccentricity (∼2 mm) for the location of the *area centralis* was also reported [17]. The possible cause for such a difference is not clear at this time.

### Susceptibility of human *fovea centralis* to retinal disease

Debilitating vision loss in human Mendelian retinopathies results from thousands of mutations in more than 200 different genes [66]. Macular degenerations is the name given to a subset of these retinopathies where the disease is primarily expressed in a 5–6 mm diameter region of the primate retina, the macula, that has the *fovea centralis* at its center. Of these, vitelliform macular dystrophy is among the most common and is caused by dominant or recessive mutations in the *BEST1* gene encoding a protein postulated to play a number of roles, including functioning as a Ca^2+^-dependent Cl^−^ channel associated with basolateral plasmalemma of the RPE [48]. The pathogenic sequence in BEST1 disease begins with defective retina-wide fluid transport across the RPE that results in a tell-tale abnormality in the electro-oculogram. Even though there is clear evidence for a retina-wide abnormality, the main disease expression however is often detected at or near the *fovea centralis* with devastating vision loss. Similarly in other macular degenerations such as those caused by *ABCA4* mutations, the abnormal gene product is known to be expressed across all rod and cone photoreceptors [67] yet the disease is initially and most severely expressed at or near the *fovea centralis*.

Other retinopathies can have more complex consequences in terms of retina-wide distribution of disease expression. For example, X-linked *RPGR* mutations can clinically manifest as forms of retinitis pigmentosa where peripheral retina is affected early and macula is generally spared, or also can manifest as macular degeneration [31], [49]. It remains mostly unknown why the macular area is susceptible to earlier and/or more severe disease expression for certain hereditary insults and why it is spared under other conditions. Likely contributors may involve retina-wide molecular gradients that may be active during retinal development which is strongly organized by the early specialization of the central retina including the *fovea centralis* and *area centralis* [10].

### Animal models of human macular degenerations

The laboratory mouse has been the pre-eminent choice of species for modeling biology and pathology of the human retina. Major advantage for the use of the mouse includes availability of advanced molecular techniques for the sophisticated manipulation of complex biological processes. An important disadvantage of mice for macular degeneration research is their natural lack of a foveo-macular region. Old-world primates on the other hand have a fovea-macular region but rarity of naturally occurring forms of macular degeneration [68]–[70], long life-spans, expense and ethical concerns make research very difficult to perform.

Large non-primate animals have become increasingly attractive to assess efficacy and safety of a variety of treatment modalities that are being considered for clinical trials in human patients [24]. Dogs, by far, have the widest array of naturally occurring inherited retinal degenerations with >18 causative genes identified to date [25]. Previously it was thought that the lack of a foveo-macular region in the canine retina would limit its use for studies aimed at understanding the pathogenesis of macular degeneration and its treatments. But our current results describing a canine retinal area with primate fovea-like cone density, and a predilection in two canine models for early disease within this area, similar to human patients, sets the stage for better understanding of the susceptibility of this region to molecularly specific insults and evaluation of potential treatments for human macular degenerations.

## Supporting Information

Figure S1 ***In vivo*** **appearance and progression of lesions in the fovea-like area of dogs with** ***BEST1*** **mutations.** (a) A well-circumscribed focal lesion is visible by near infra-red (NIR) confocal scanning laser ophthalmoscopy (cSLO) in the fovea-like area of each retina of a 17 week-old dog. Cross-section imaging of the retina by optical coherence tomography (OCT) through these lesions (green horizontal arrows) shows focal retinal detachments (white arrows). (b) cSLO/OCT imaging of the retina of a 35 week-old dog shows a large focal retinal detachment with a characteristic hyperfluorescent pseudohypopyon (white arrow) on autofluorescence (AF) imaging. The pseudohypopyon lesion corresponds to the subretinal material of increased reflectivity detectable on the vertical OCT scan (white arrow). (c) cSLO/OCT longitudinal imaging in another *BEST1* mutant dog shows at 26 weeks of age a discrete retinal elevation (black arrow) within the fovea-like area at the site where the outer nuclear layer (ONL) is the thinnest. A clear retinal detachment with accumulation of autofluorescent material is seen at 98 weeks of age (white arrow).
(TIF)

Table S1 **Histological characteristics of the fovea-like area of normal dogs.**
(DOCX)

Table S2 **Age of occurrence of initial lesions of the fovea-like area and secondary extra foveal lesions in** ***BEST1*** **mutant dogs.**
(DOCX)

Table S3 **Characteristics of the fovea-like area in** ***RPGR*** **mutant dogs.**
(DOCX)

Video S1 **Two photon microscopy imaging of the fovea-like area of a 24 week-old wildtype dog.** The cone insoluble extracellular matrix domain was labeled with PNA lectin (red), retinal ganglion cells (RGCs) with Brn3a antibody (red), retinal blood vessels with von Willebrand factorVIII (green), and nuclei with DAPI (blue).
(WMV)

Video S2 **3D rendering of the fovea-like area of a 6.4 year-old wildtype dog imaged by 2 photon microscopy.** The cone insoluble extracellular matrix domain was labeled with PNA lectin (red), retinal ganglion cells (RGCs) with Brn3a antibody (red), retinal blood vessels with von Willebrand factorVIII (green), and nuclei with DAPI (blue).
(WMV)
