# Supplementary figures and images for: Canine Retina Has a Primate Fovea-Like Bouquet of Cone Photoreceptors Which Is Affected by Inherited Macular Degenerations
Source: PLoS One. 2014 Mar 5;9(3):e90390. doi: 10.1371/journal.pone.0090390 (PMC3944008; doi:10.1371/journal.pone.0090390)

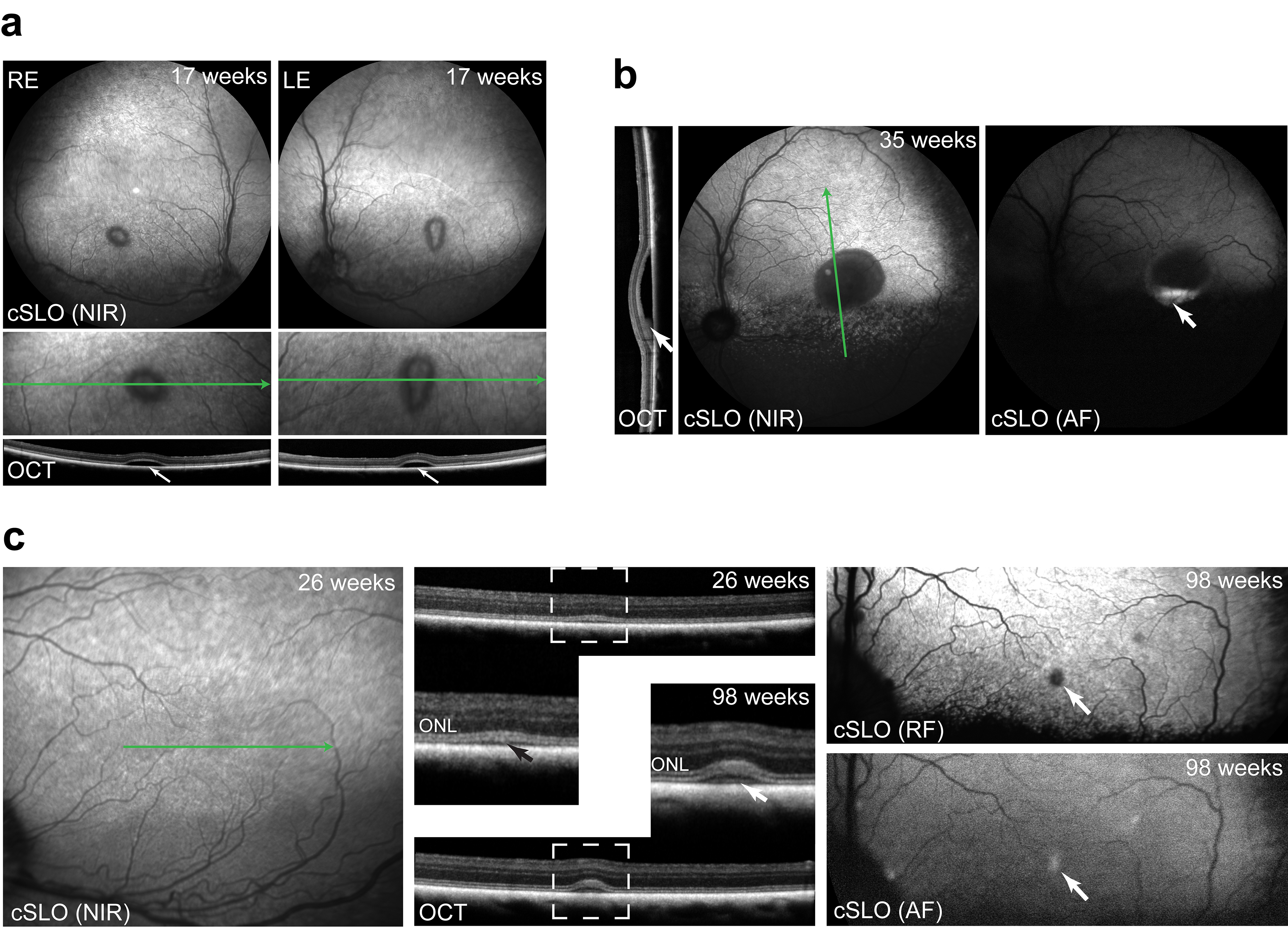

Supplement: Figure S1 — In vivo appearance and progression of lesions in the fovea-like area of dogs with BEST1 mutations. (a) A well-circumscribed focal lesion is visible by near infra-red (NIR) confocal scanning laser ophthalmoscopy (cSLO) in the fovea-like area of each retina of a 17 week-old dog. Cross-section imaging of the retina by optical coherence tomography (OCT) through these lesions (green horizontal arrows) shows focal retinal detachments (white arrows). (b) cSLO/OCT imaging of the retina of a 35 week-old dog shows a large focal retinal detachment with a characteristic hyperfluorescent pseudohypopyon (white arrow) on autofluorescence (AF) imaging. The pseudohypopyon lesion corresponds to the subretinal material of increased reflectivity detectable on the vertical OCT scan (white arrow). (c) cSLO/OCT longitudinal imaging in another BEST1 mutant dog shows at 26 weeks of age a discrete retinal elevation (black arrow) within the fovea-like area at the site where the outer nuclear layer (ONL) is the thinnest. A clear retinal detachment with accumulation of autofluorescent material is seen at 98 weeks of age (white arrow). (TIF) [file pone.0090390.s001.tif]
