# Supplementary material for: Canine Retina Has a Primate Fovea-Like Bouquet of Cone Photoreceptors Which Is Affected by Inherited Macular Degenerations
Source: PLoS One. 2014 Mar 5;9(3):e90390. doi: 10.1371/journal.pone.0090390 (PMC3944008; doi:10.1371/journal.pone.0090390)
Supplement: Table S1 — Histological characteristics of the fovea-like area of normal dogs. (DOCX) [file pone.0090390.s002.docx]

**Table S1**. **Histological characteristics of the fovea-like area of normal dogs.**

| Dog ID | Age | Eye | | Tissue process (cone label) | Distance from ONH edge  (μm) | Length  (μm) | Area  (μm^2^) | Max # of rows of cones | Max # of rows of rods | Max # of rows of RGCs | Cephalic Index (%) | Cone density  (cells/mm^2^) |
| --- | --- | --- | --- | --- | --- | --- | --- | --- | --- | --- | --- | --- |
|  | | |  | | | | | | | | | |
| I514 | 2 wks | RE | | C.S. (hCA) | 3,400 | 62 | - | 3 | 1.5 | 3 | - | 90,141 |
| SPF3 | 3 wks | RE | | C.S. (hCA) | - | 106 | - | 3 | 1 | 3 | - | 78,873 |
| SPF4 | 4 wks | RE | | C.S. (hCA) | 3,650 | 86 | - | 2 | 3 | 3 | - | 135,265 |
| AS277 | 6 wks | LE | | C.S. (hCA) | 4,000 | 111 | - | 3 | 2 | 2.5 | - | 133,333 |
| EM249 | 7 wks | RE | | C.S. (hCA) | 4,200 | 92 | - | 3 | 1.5 | 3 | - | 132,352 |
| E1058 | 7 wks | * | | W.M. (hCA) | 3,250 | - | 15,425 | - | - | - | 50 | 63,800 |
| N240 | 12 wks | LE | | C.S. (hCA) | 3,718 | 142 | - | 3 | 1.5 | 3 | - | - |
| D204 | 21 wks | RE | | C.S. (hCA) | 3,600 | 106 | - | 3 | 4.5 | 2.5 | 60 | 154,000 |
| CEACGN | 24 wks | LE | | W.M. (PNA) | 4,000 | - | 11,592 | - | - | - | 57 | 136,400 |
| CDJCCJ | 35 wks | LE | | W.M. (PNA) | 3,750 | - | 17,907 | - | - | - | 57 | 75,600 |
| GI98 | 35 wks | LE | | C.S. (hCA) | 3,700 | 116 | - | 3.5 | 3 | 2.5 | 57 | 212,121 |
| D312 | 95 wks | * | | W.M. (PNA) | 3,800 | - | 8,637 | - | - | - | 60 | 119,600 |
| N269 | 4.75 yrs | LE | | C.S. (hCA) | 4,290 | 131 | - | 4 | 2 | 3 | 57 | 157,205 |
| N237 | 5.5 yrs | RE | | C.S. (hCA) | 4,247 | 102 | - | 2.5 | 5 | 3 | - | 172,414 |
| LD101 | 6.8 yrs | * | | W.M. (PNA) | 3,700 | - | 23,672 | - | - | - | - | 114,400 |
| BR217 | 8 yrs | RE | | C.S. (hCA) | 3,600 | 194 | - | 3 | 3 | 2.5 | 50 | 124,481 |
|  | | |  | | | | | | | | | |
| **MEAN** | | | | | **3,794** | **113** | **15,447** | **2.9** | **2.6** | **2.8** | **56** | **126,666** |
| **SD** | | | | | **302** | **34** | **5,808** | **0.6** | **1.2** | **0.3** | **3.9** | **39,374** |
|  | | |  | | | | | | | | | |
| E1058 | 7 wks | RE | | W.M. (hCA) | 3,100 | - | 14,432 | - | - | - | 50 | 67,600 |
|  |  | LE | | W.M. (hCA) | 3,400 | - | 16,418 | - | - | - |  | 60,000 |
| D312 | 95 wks | RE | | W.M. (PNA) | 3,600 | - | 8,017 | - | - | - | 60 | 122,000 |
|  |  | LE | | W.M. (PNA) | 4,000 | - | 9,257 | - | - | - |  | 117,200 |
| LD101 | 6.8 yrs | RE | | W.M. (PNA) | 3,700 | - | 20,970 | - | - | - | - | 112,800 |
|  |  | LE | | W.M. (PNA) | 3,700 | - | 26,375 | - | - | - |  | 116,000 |

LE: left eye; RE: right eye; C.S.: cryosection; W.M. wholemount; RGC: retinal ganglion cell; hCA: human cone arrestin antibody; PNA: peanut agglutinin
*: Mean of RE and LE so as to be weighted as one individual. Specific values for each eye are shown below.
