# Supplementary material for: Canine Retina Has a Primate Fovea-Like Bouquet of Cone Photoreceptors Which Is Affected by Inherited Macular Degenerations
Source: PLoS One. 2014 Mar 5;9(3):e90390. doi: 10.1371/journal.pone.0090390 (PMC3944008; doi:10.1371/journal.pone.0090390)
Supplement: Table S2 — Age of occurrence of initial lesions of the fovea-like area and secondary extra foveal lesions in BEST1 mutant dogs. (DOCX) [file pone.0090390.s003.docx]

**Table S2**. **Age of occurrence of initial lesions of the fovea-like area and secondary extra foveal lesions in *BEST1* mutant dogs.**

| **Dog ID** | **Genotype** | **1^st^ detection of retinal separation  at the fovea-like area (age in weeks)** | **1^st^ detection of autofluorescence  at the fovea-like area**  **(age in weeks)** | **1^st^ detection of extra foveal lesions**  **(age in weeks)** |
| --- | --- | --- | --- | --- |
|  |  |  |  |  |
| EM286 | 1/1 | 52 (BE) | ND | ND |
| EM322 | 1/1 | 26 (BE) | 98 (BE) | 39 (RE); 75 (LE) |
| EM341 | 1/1 | 42 (RE); 62 (LE) | ND | ND |
| EM385 | 1/1 | 17 (BE) | 35 (BE) | 35 (BE) |
| EM386 | 1/1 | 17 (BE) | 37 (LE); ND (RE)* | ND |
| EM356 | 1/3 | 12 (BE) | 30 (BE) | 47 (RE); ND (LE) |
| LH21 | 3/3 | 10 (BE) | 21 (BE) | 21 ( BE) |

Genotype: 1/1-homozygous mutant for c.73C>T; 3/3-homozygous mutant for the c.[1388delC; 1466G>T] linked mutations; 1/3-compound heterozygous for the c.73C>T and the c.[1388delC; 1466G>T] mutations; LE: left eye; RE: right eye; BE: both eyes; ND: not detected; *: included in gene therapy study.
