# Supplementary material for: Canine Retina Has a Primate Fovea-Like Bouquet of Cone Photoreceptors Which Is Affected by Inherited Macular Degenerations
Source: PLoS One. 2014 Mar 5;9(3):e90390. doi: 10.1371/journal.pone.0090390 (PMC3944008; doi:10.1371/journal.pone.0090390)
Supplement: Table S3 — Characteristics of the fovea-like area in RPGR mutant dogs. (DOCX) [file pone.0090390.s004.docx]

**Table S3**. **Characteristics of the fovea-like area in *RPGR* mutant dogs.**

| **Dog ID** | **Age**  **(weeks)** | **Eye** | **Tissue process**  **(cone label)** | **Distance from ONH (μm)** | **Max # of rows of cones** | **Max # of rows of rods** | **Max # of rows of RGCs** |
| --- | --- | --- | --- | --- | --- | --- | --- |
|  |  |  |  |  |  |  |  |
| Z215 | 2 | LE | Epon plastic (aII-m./PPDA) | 3,700 | 2 | 2 | 3 |
| Z266 | 4 | LE | Cryosection (CA) | 3,650 | 2 | 1 | 3 |
| Z254 | 7 | LE | Cryosection (CA) | 3,500 | 2 | 1 | 3 |
| Z208 | 20 | LE | Epon plastic (aII-m./PPDA) | 3,700 | 2 | 0-1 | 3 |
| Z377 | 22 | RE | Cryosection (CA) | ND | 2 | 0-1 | 3 |
| EMZ6 | 145 | RE | Cryosection (H&E) | ND | 1 | 0 | 2 |
|  |  |  |  |  |  |  |  |

LE: left eye; RE: right eye; RGC: retinal ganglion cell; (aII-m./PPDA): azure II-methylene blue/paraphenylenediamine;
CA: human cone arrestin antibody; H&E: hematoxylin and eosin; ND: not determined.
